# The carcinogenic PAHs in breads, amount, analytical method and mitigation strategy, a systematic review study

**DOI:** 10.1186/s12889-024-18413-0

**Published:** 2024-06-07

**Authors:** Gholamreza Tavoosidana, Mansoreh Abdolhosseini, Yeghaneh Mazaheri, Burhan Basaran, Parisa Shavali-gilani, Parisa Sadighara

**Affiliations:** 1 Molecular Medicine Department, School of Advanced Medical Technologies, Tehran University of Medical Sciences, Tehran, Iran; 2 Department of Environmental Health, Food Safety Division, School of Public Health, Tehran University of Medical Sciences, Tehran, Iran; 3 Department of Nutrition and Dietetics, Faculty of Health Sciences, Recep Tayyip Erdogan University, Rize, 53100, Turkey

**Keywords:** Bread, PAHs, Carcinogen, Analytical method, Risk

## Abstract

Bread is one of the most consumed foods all over the world. Several contaminants are identified in bread. Polycyclic aromatic hydrocarbons (PAHs) is one of these contaminants. This systematic study evaluates the amount of four carcinogenic PAHs (PAH4) in various types of breads. To conduct this study, a comprehensive search was carried out using keywords of polycyclic aromatic hydrocarbons, PAHs, PAH4, and bread, with no time limitations. 17 articles were selected and fully evaluated. The observed range of PAH4 concentrations in bread varied from non-detected (ND) to 20.66 µg/kg. In the sample preparation process for analysis, an ultrasonic bath was predominantly utilized. Most chromatographic methods are able to measure PAHs in food, but the GC-MS method has been used more. To mitigate PAH levels in bread, it is suggested to incorporate antioxidants during the bread-making process. Furthermore, the type of bread, the type of fuel used to bake the bread, the temperature and the cooking time were some of the factors affecting the amount of PAH. Restricting these factors could significantly reduce PAH content. Regarding the risk assessment conducted in the manuscript, it was determined that industrial breads are usually considered safe. However, some traditional breads may pose risks in terms of their potential PAH content.

**Supplementary Information:**

The online version contains supplementary material available at 10.1186/s12889-024-18413-0.

## Introduction

Certain carcinogenic compounds are an integral part of food and water [1]. Among these, polycyclic aromatic hydrocarbons (PAHs) are carcinogenic compounds that are produced due to incomplete combustion and lead to environmental pollution [2, 3]. In some cases, it is formed as a result of pyrolysis of organic matters [3]. Epidemiological evidence in specific geographical areas has confirmed the association between PAHs and certain cancers, notably esophageal cancers [4]. The compounds are non-polar and lipophilic and easily pass through cell membranes and body barriers. PAH metabolites are active compounds that have the ability to attack macromolecules, including DNA [5]. They also lead to malformation [6]. Additionally, a confirmed link exists between PAHs and an elevated risk of diabetes [7].

Most human exposure to PAHs occurs through food consumption [8–10]. PAHs can contaminate food during processing and cooking through the pyrolysis of organic compounds like fat and protein. Environmental factors, such as soil and water contamination, also contribute to the presence of PAHs in food [11]. Irrigation of plants with PAHs contaminated water is another cause of contamination [12].

Bread is a major source of energy and is the most consumed per capita among food items [13]. Bread can be contaminated by PAHs from its raw materials such as flour and during the baking process [13]. PAHs are divided into heavy and light PAHs. PAHs up to four rings are light and more than that are heavy PAHs. Heavy PAHs are more toxic and more stable [14].

In the past, Benzo[a]pyrene as one of the heavy PAHs was considered as a carcinogenic indicator for food contamination with PAHs [5]. This compound is genotoxic [15]. However, current practices have shifted towards utilizing PAH4, a combination of benzo[b]fluoranthene(B[b]F), chrysene (Chr), benzo[a]pyrene(B[a]P), benzo[a]anthracene(B[a]A) as the preferred indicator [15]. The European Union introduces PAH4 as reasonable markers for the contamination of PAHs in food [16]. These authorities have announced permissible limits for both PAH4 and benzo[a]pyrene in some foods. The competent authorities of China have announced a permissible limit for benzo[a]pyrene in food [17].

These four PAHs are genotoxic, mutagenic and carcinogenic. According to the International Agency for Research on Cancer; Benzo[a]pyrene is included in category 1(carcinogenic to humans) and benzo[b]fluoranthene, chrysene, benz[a]anthracene belong to category 2B (possibly carcinogenic to humans) [18]. This study was designed to assess the risk associated with PAH4 and its levels in different types of bread. Factors affecting PAH concentration in bread samples are also discussed. In addition, this research provides comprehensive information about the sample preparation process, with the aim of ensuring accuracy and precision in measurements.

## Methods

### The inclusion and exclusion criteria

The inclusion criteria for this study comprised research manuscripts specifically measuring the quantity of PAH4 in bread. The aim of this study was to evaluate carcinogenic PAHs in bread. In this regard, PAH4 was a more valid factor than Benzo[a]pyrene. Therefore, PAH4 was chosen as an index and inclusion criterion in this systematic study. Review manuscripts, book chapters, experimental and research studies, and other food products other than bread were excluded from this systematic review. Manuscripts that declared the amount of PAH4 were included in the study. In addition, the factors affecting the formation of PAHs in breads were investigated. Various methods of sample preparation and analytical methods were considered, and their data were extracted. Also, in this systematic study, existing methods for reducing PAHs level in bread were also evaluated.

### Search strategy

The systematic review’s search was conducted on July 20, 2023, utilizing the keywords including PAHs or PAH4 and polycyclic aromatic hydrocarbons and bread. The chosen databases for the search were PubMed, Scopus, and Science Direct. Regrettably, access to the Embase database was not feasible for the research team. In order to perform the search, at first, the PubMed database and the Google scholar search engine were checked to see if there was any systematic research in this regard. Due to the fact that no related research had been done, this search was conducted without time limit. The search was done by two authors independently (M.A and G.T). Both results were the same.

## Results

### The result of search

**Fig. 1:**
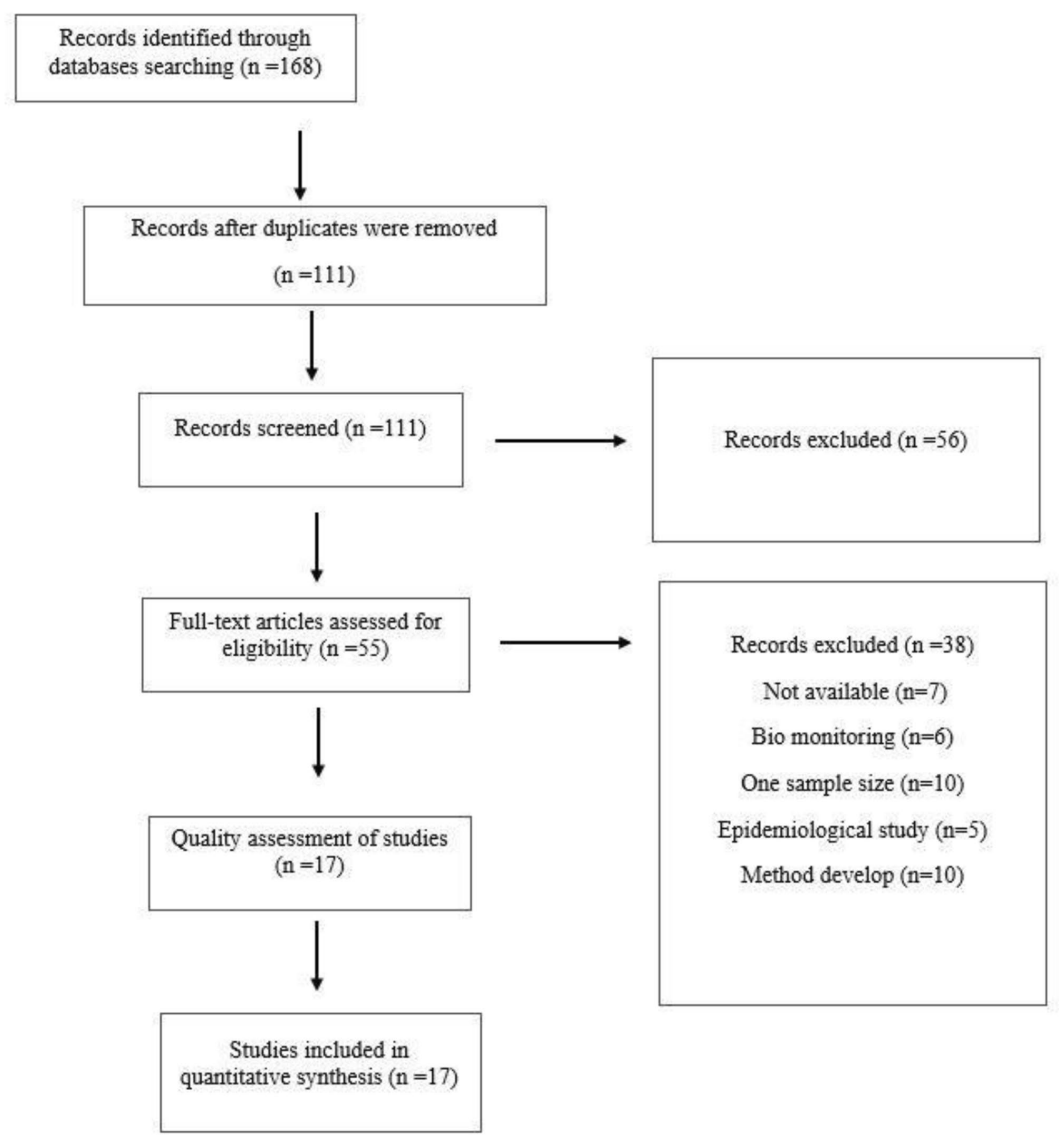
The diagram of study

After searching, 168 manuscripts were entered into the Endnote software (Fig. 1). Duplicate manuscripts were removed. Initially, the title and abstract of the articles were scrutinized. The title and abstract of the manuscripts were reviewed according to the inclusion criteria of this research. 27 manuscripts were related to laboratory research and investigation of toxicity mechanism. 19 manuscripts focusing on the measurement of PAHs in other foods were evaluated. 10 manuscripts were review papers and book chapters that were excluded from this research according to the exclusion criteria. 55 articles were selected for comprehensive evaluation. The assessment of these articles was based on five criteria outlined in the protocol. The data of the articles that scored 3 or more in these criteria were extracted. The criteria for evaluating the manuscript included using a valid method for measurement, high and acceptable number of samples, clear disclosure of sample preparation details, announcing the exact details of the analytical method, and announcing the amount of PAH4 measured along with its unit on real samples. It is important to note that some manuscripts did not assess real samples and instead focused on method development.

### Data extraction

Seventeen manuscripts that both satisfied the inclusion criteria and achieved the required score in the qualitative evaluation were chosen for data extraction. Extracted information included the amount of PAH4, number of samples, analytical method, how to prepare the samples, type of bread, Recovery percent, risk assessment, country and the name of the first author, and the year of publication of the manuscripts. It is noteworthy that the majority of the studies reported the measured values, with the exception of the study of Gong et al., which presented the results graphically.

**Table 1:** Extracted data based on study inclusion criteria

Analytical method	Sample preparation	Recovery percent	Risk assessment	Amount	Author /year /Country
GC-MS/MS	Extraction: Absorbing moisture with anhydrous sodium sulfate, using two solvents dichloromethane/hexane, sonicate, evaporation of solvents, regeneration of fat with cyclohexane/ethyl acetate mixture, evaporation of solvents, addition of cyclohexane to the dried residue Clean up: SPE	In range:92–103%	-	Rye bread: 0.31–1.62 Wheat bread: 0.22–1.62 µg/ kg	Rozentale/ 2016 Latvia
GC-MS	Digestion: Using microwave Extraction: adding NaCl, and using two solvents (acetone/ tetrachloroethylene)	-	Dietary intakes of PAH4(traditional bread): 2.53 Dietary intakes of PAH4(Industrial bread): 1.54 ng/person/day	Baguette 0.48–12.55 µg/ kg	Kamalabadi/ 2018 Iran
				Lavash 0.48–4.94 µg/ kg	
				Taftoon 0.48–20.66 µg/ kg	
GC-MS	Extraction: Dissolve in n-hexane and place in ultrasonic bath, absorbing moisture with anhydrous sodium sulfate, evaporation of solvent, addition of cyclohexane to the dried residue Clean up: SPE	B[b]F:88% Chr:86% B[a]P:91% B[a]A:88%	-	*N* = 20 0.28 ± 0.09 µg/ kg	Kacmaz/ 2016 Turkey
GC-MS	Extraction: Dissolve in n-hexane and polyacrylic acid, absorbing moisture with anhydrous sodium sulfate, evaporation of solvent, addition of cyclohexane to the dried residue, Clean up: SPE	B[b]F:88% Chr:97% B[a]P:93% B[a]A:101%	-	0.11–0.22 *N* = 20 µg/ kg	Kacmaz/ 2016 Turkey
GC-MS	Extraction: Dissolve in dichloromethane/hexane in an ASE, Clean up: SPE	In range:71.5–96.5%	-	ND *N* = 4 LOQ(µg /kg) In range: 0.1 to 0.7	Iwegbue/ 2016 Nigeria
HPLC–FLD/DAD	Extraction: Dissolve in n-hexane /acetone and place in ultrasonic bath, absorbing moisture with anhydrous sodium sulfate, evaporation of solvent, addition of cyclohexane/ethyl acetate to the dried residue Clean up: using gel permeation chromatography	B[b]F:80.6% Chr:81.2% B[a]P:106% B[a]A:83.7%	MOE (PAH4): 198,100	Wheat-rye bread: 0.05– 0.47, rye bread: 0.23–0.45 whole meal rye bread: 0.21–1.29 µg/ kg	Ciecierska/ 2013 Poland
GC-MS	QuEChERS method was used for extraction. Mixing with acetonitrile and sodium sulfate, centrifugation, evaporation of solvents, regeneration with MeCN, MgSO_4_, and PSA	B[b]F:93.3% Chr:100% B[a]P:90.2% B[a]A:86.7%	-	Taftoon ND *N* = 72 LOQ(ng/g) B[b]F = 1.1 Chr = 0.6 B[a]*P* = 2.5 B[a]A = 0.8	Moradi/2020 Iran
GC-MS/MS	Extraction: Dissolve in dichloromethane/hexane in an ASE, extracting with hexane/acetone Clean up: SPE	In range:50–120%	MOE (PAH4): 42,000	Lower than LOD One sample: 1.4 µg/ kg *N* = 35	Hokkanen/ 2021 Finland
GC-MS	Extraction: Reflux with ethanolic solution of KOH Clean up: column chromatography	-	HQ(B[a]P) for a sample higher than one	Mean:7.3 µg/ kg	Orecchio/ 2009 Italy
GC-MS	QuEChERS method was used for extraction. Mixing with acetonitrile and sodium sulfate, centrifugation, evaporation of solvents, regeneration with MeCN, MgSO_4_, and PSA	B[b]F:99% Chr:100% B[a]P:90.7% B[a]A:93.1%	-	Bread (Sangak) ND *N* = 47 LOQ(ng/g) B[b]F = 1.1 Chr = 0.6 B[a]*P* = 2.5 B[a]A = 0.8	Peiravian/ 2020 Iran
HPLC–FLD	Studying belongs to the distant past. Details are not stated. Hexane was used for extraction	-	-	Mean:0.49 µg/ kg *N* = 3	Dennis/1991 UK
GC-FID	Extraction: Dissolve in acetone and methylene chloride and place in ultrasonic bath	-	Dietary intakes of B[a]P: in range of 0.01 to 0.19 µg/kg	Mean:1.07 µg/ kg *N* = 20	Udowelle/ 2017 Nigeria
GC-MS	Extraction: Using n-hexane solvent in an ultrasound bath, evaporation of solvents, regeneration with acetonitrile/acetone mixture, evaporation of solvents, addition of n-hexane to the dried residue Clean up: SPE	In range:68.9-106.2%	-	Bread youtiao 3.20 ± 0.45	Li/2016 China
HPLC	Extraction: Using n-hexane solvent in an ultrasound bath, evaporation of solvents, regeneration with n-hexane Clean up: Sep-pack silica and SPE	-	-	Toasted bread ND *N* = 3	Salgueiro/ 2007 Spain
				Electric oven bread ND *N* = 3 LOQ(µg/ kg) B[b]F = 0.25 Chr = 0.7 B[a]*P* = 0.07 B[a]A = 0.1	
GC-MS	Extraction: Using n-hexane solvent in an ultrasound bath (for 3 time), evaporation of solvents, regeneration with acetonitrile/acetone mixture, evaporation of solvents, addition of n-hexane to the dried residue Clean up: SPE	In range:71–108%	-	Bread youtiao The results were displayed as a graph	Gong/2017 China
HPLC–FLD	Extraction: Using n-hexane solvent in an ultrasound bath(for 2 times), evaporation of solvents, regeneration with n-hexane Clean up: Sep-pack silica	B[b]F:92% Chr:85% B[a]P:103% B[a]A:82%	-	Smoked bread Mean = 0.20 µg/ kg *N* = 5	Fasano/2016 Italy
GC-MS	QuEChERS method was used for extraction. Mixing with acetonitrile and sodium sulfate, centrifugation, evaporation of solvents, regeneration with MeCN, MgSO_4_, and PSA	In range:86–111%	-	Barbary bread ND *N* = 40 LOQ (ng/g) B[b]F = 1.1 Chr = 0.6 B[a]*P* = 2.5 B[a]A = 0.8	Moradi/2019 Iran

Note: ND; not detected, QuEChERS; quick easy cheap effective rugged safe, ASE; accelerated solvent extractor, SPE; solid-phase extraction, PSA; primary secondary amine, MOE; margin of exposure, B[b]F; benzo[b]fluoranthene, Chr; chrysene, B[a]P; benzo[a]pyrene, B[a]A; benzo[a]anthracene, MeCN; acetonitrile, LOQ; limit of quantification

## Discussion

Bread is culturally and economically important and valuable [19]. It provides nutrients such as vitamins, iron, protein, and calcium [20]. On average, a person consumes 70 kg of bread per year [21]. Its fundamental ingredients include flour, yeast, salt, and water [21]. Benzo[a]pyrene was often used to evaluate the carcinogenic PAHs. But the European Union announced that the four PAHs (PAH4) B[b]F, Chr, B[a]P, B[a]A gave a more accurate assessment than Benzo[a]pyrene alone [16]. Bread is one of the main components of diets around the world. Therefore, its safety is important and is necessary to evaluate the amount of contaminants in bread regularly. Accurate methods are imperative for precise assessments of contaminant quantities. PAHs are present in low amounts in bread products, so it is necessary to extract and prepare samples carefully [22]. Moreover, determining the levels of carcinogenic PAHs in different bread types contributes to assessing the associated risks. For this purpose, this systematic study was designed.

### Amount and factors affecting the amount of toxin in breads

According to the European Union, the limit of PAH4 in cereal-based products is 1 µg/kg (commission regulation (EU) No 835/2011). The amount of PAH4 between ND (not detected) and 20.66 µg/ kg was observed among the extracted data (Table 1). According to the extracted data, high levels of PAH4 were observed in the bread samples that were prepared in the traditional way. The amount of PAH formation in food depends on the temperature and duration of cooking [23]. There are studies proving that cooking conditions affect the formation of PAHs in foods [24].. According to studies, the amount of PAHs in traditional bread is higher than industrial bread [25]. The temperature can be controlled in industrial baking.

In a study, it was found that carcinogenic PAHs exceeded the EU limit in 14% of the analyzed bread samples [13]. In the studies of Iwegbue et al. and Peiravian et al., the levels of PAH4 in bread were reported ND [26, 27]. Additionally, the research conducted by the same team on Sangak bread revealed no detection of PAH4 [27]. Moreover, in the majority of samples in the study by Hokkanen et al., the reported amount of PAH4 was non-detectable (ND) [28]. In this study, the lowest MOE (Margin of Exposure) value determined among food items was calculated for bread [28]. In the study of Udowelle et al., the average amount of PAH4 was reported as 1.07 µg/kg [29]. Olive oil is used in the composition of the tested breads, and it is possible that some of the contamination is related to olive oil. The presence of PAHs in vegetable oils, including olive oil, has been extensively studied. Contamination can occur during cultivation and the oil production process. Factors such as the use of solvents, detergents, and lubricants during oil processing may lead to PAH contamination. Even the packaging of vegetable oils, such as polyethylene packaging, can be a potential source of PAH contamination [30].

In a research study, the evaluation of PAH4 levels in three smoked products smoked bread, smoked cheese, and smoked meat revealed that the lowest amount was found in smoked bread [31]. Smoking is one of the traditional methods of food preparation. During smoking, phenolic compounds, which play an important role in the taste of food, are produced [32]. However, it is important to note that, along with these compounds, toxic substances such as PAHs are also produced during the smoking process. In a study, BaP was observed in 100% of smoked cheeses [33]. Additionally, in another study, the amount of PAH4 in smoked meat products was in the range between 0.15 and 34.65 µg /kg [32]. The amount of PAHs in smoked products depends on the time and temperature during processing, and the amount of moisture and the type of wood used in smoking [32]. As a rule, smoked bread is made in a shorter time than other smoked products. Also, the amount of PAHs depends on fat and lipid [34]. This can be explained by the lower amount of PAHs in smoked bread compared to other smoked products such as meat and cheese.

In the study conducted by Kamalabadi et al., the amount of PAHs in three types of baguette, lavash and taftoon bread was measured. The amount of PAH4 in all three types of bread was different and the authors stated this difference resulted from temperature and baking time [35]. In the study of Moradi et al., the amount of PAH4 in Taftoon and Barbary bread samples was not detected [36, 37]. However, the range of 0.48–20.66 µg/kg was reported in another study. This difference in quantity probably depends on the cooking time and temperature. In the study of Moradi, the baking temperature was 216–300 °C for taftoon bread and 170–250 °C for Barbary bread [36, 37]. In the study of Kamalabadi et al., it was announced for 2.5 min at a temperature of 310 °C. Furthermore, in the study of Ciecierska et al., the level of PAH4 in three types of bread was investigated. The highest amount was observed in whole meal rye bread. This difference was due to the difference in baking temperature. The baking temperature of this type of bread was 20 °C higher than the other two types of bread [3]. Therefore, temperature and cooking time are direct and strong influencing factors in the amount of PAH4.

The type of fuel used in cooking has a notable impact on the levels of PAHs. In the study conducted by Salgueiro et al., the measurement of PAH4 was carried out on two types of toast bread prepared using charcoal and an electric oven. The toast bread cooked with charcoal was subjected to a temperature of 300 °C, while the bread baked in an electric oven was prepared at 200 °C. Interestingly, PAH4 was not detected in the toast bread baked in the electric oven. In contrast, a significant amount of Benzo[a]pyrene (0.50 ± 0.25 µg/kg) was observed in the bread prepared using charcoal. This finding emphasizes the influence of the cooking method and fuel type on the presence of PAHs in food products [5].

Similar to these results, in a study, some carcinogenic PAHs were investigated in toast, and the majority of them were reported as ND. The process of making toast typically involves lower temperatures, and commercial toast bread is commonly prepared within the temperature range between 220 and 250 °C [38]. In one study, only benzo[a]pyrene was reported in toasted bread [39].

Additionally, the type of fuel used significantly affects the levels of PAHs. Toast bread, in particular, tends to have lower amounts of PAHs due to its use of indirect heat sources [40]. Bread baked using electricity has less PAHs [41]. Bread that is baked on wood or using diesel fuel or solar fuels has very high levels of PAHs. In previous studies, there was no difference in the amount of PAHs between bread made with gas and electricity [42]. Moreover, in studies where the breads were prepared in an industrial process, PAH4 levels were reported to be lower compared to bread prepared in a traditional manner and using wood as a fuel source. In the study conducted by Orecchio et al., bread prepared using wood as a fuel source was found to have elevated levels of PAH4 [43]. Wood was used as fuel to make bread in this study [43]. Similarly, in India, the investigation of PAH4 levels in two types of bread, Tawa and Tandoori, revealed significantly higher levels of Benzo[a]pyrene in Tandoori bread. According to risk assessment, the carcinogenicity of Tandoori bread was 3.7 times higher than that of Tawa bread. Tandoori bread is baked directly in a tandoor. Baking this bread in the vicinity of coal leads to significant production of PAHs [44]. The amount of PAHs in the bread prepared in wood flame reached 350 µg/ kg [45]. In a study conducted in Spain, the amount of PAH4 in bread prepared in a wooden oven was investigated [31]. The amount of PAH4 was measured relatively low and around 0.2 µg/ kg. Because these breads were not cooked in the flame and were far from the flame [31].

Other factors affecting the amount of PAHs in bread are type of bread and the amount of PAHs in the raw material [34]. 78% of consumed bread is based on wheat [46]. The type of bread has an effect on the amount of PAHs. In the study of Rozentale et al., the amount of PAH4 in Rye bread was higher than wheat bread [13]. The amount of PAHs in bran/granary breads was higher than white breads [34]. Most studies suggest that the amount of PAHs in wheat-based breads is generally lower than that in barley and rye-based bread [13, 47]. Research indicates that PAHs with low molecular weights tend to be absorbed more by wheat plants from the soil [48]. Furthermore, multicereal bread has a higher PAHs content. Youtiao is bread fried in oil. It is a traditional type of bread for thousands of years in China [49]. The amount of PAH4 in this bread is more than the usual bread [49]. Similarly, Tah-dig, a product where bread is fried in oil, exhibits significant amounts of detected PAHs [50]. It is probably the frying process that leads to a significant increase in PAH in these two types of bread.

Another factor affecting the amount of PAHs is the amount of fat in the bread. The higher the amount of fat is, the higher the amount of PAHs is. This is due to the pyrolysis of fats. Furthermore, the amount of PAHs in food is directly related to their fat content [51]. Also, bread made from oats has higher fat [21]. Usually, the amount of PAHs in oat bread is higher than in other types of bread.

**Fig. 2:**
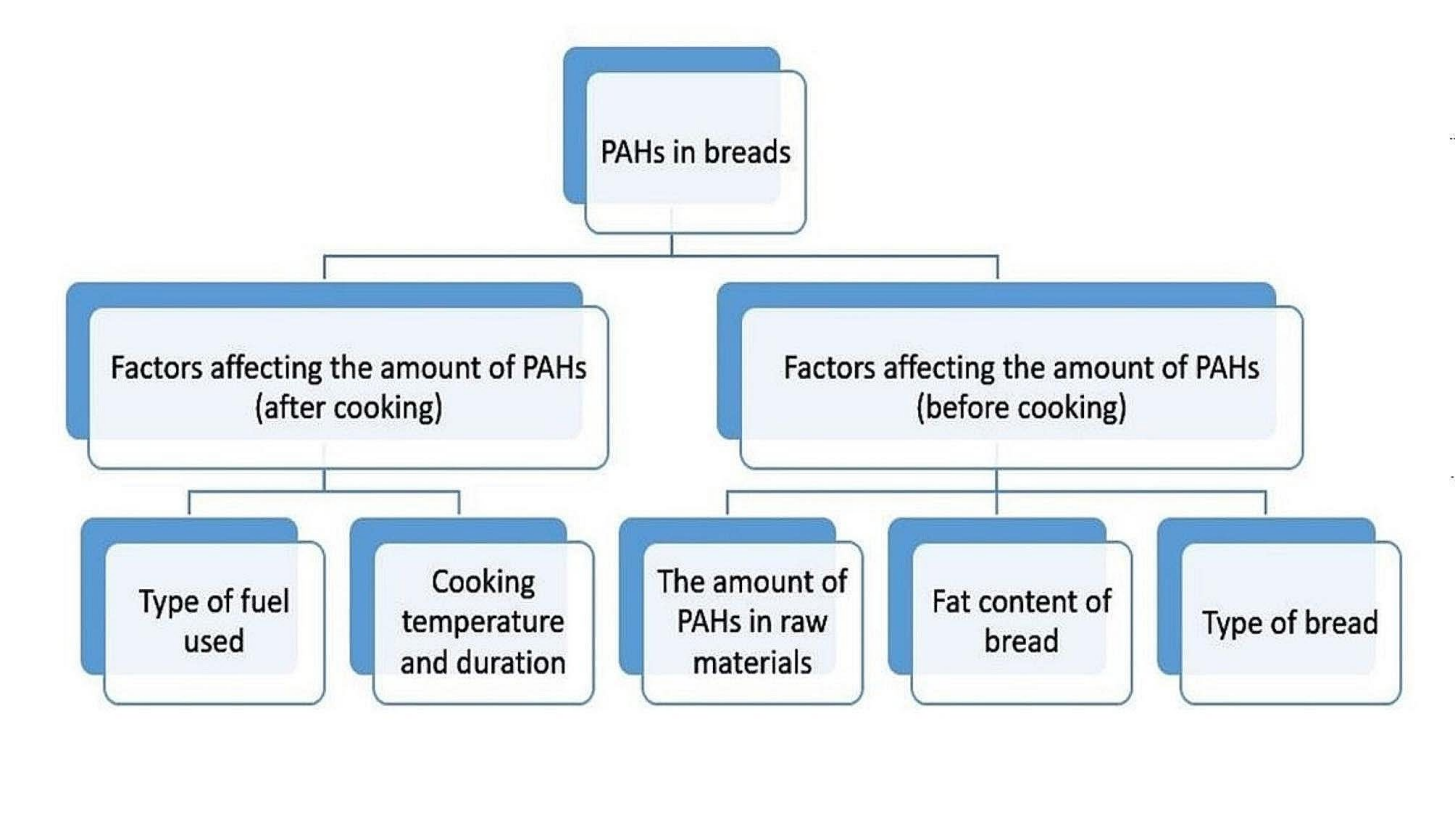
Diagram of factors affecting the amount of PAHs in food

In general, the amount of PAHs is affected both during processing and cooking stages, as well as the initial PAH content in the raw materials. It is possible that the amount of PAHs in the bread is caused by the amount of PAHs in the raw materials of the bread. Plants can absorb soil PAHs. Plants that are cultivated longer will have more PAHs [52]. The influencing factors in the formation of PAHs in bread are summarized in Fig. 2.

In only one study, the method of reducing PAHs in bread was discussed. Three natural antioxidants of rosemary extract, tea and bamboo were added to a type of bread called youtiao. Bamboo and rosemary significantly reduced the amount of PAHs [53].

### Risk assessment

The data pertaining to risk assessment were extracted from studies in which the carcinogenic risk was calculated. The Carcinogenic risk of compounds is assessed using the Margin of Exposure (MOE) factor, determined by the formula: MOE = BMDL_10_/EDI. EDI is estimated daily intake. BMD (benchmark dose) is an alternative strategy for calculating tolerable daily intake (TDI) [54].

According to the study results, BMDL10(incidence of 10% of induced tumor) for PAH4 is calculated to be 0.34 mg/kg bw/day [55]. If the MOE value is less than 10,000, there is a risk of carcinogenesis. A study conducted in Poland calculated this value as 198,100, indicating the safety of bread concerning the risk of carcinogenicity of PAH4 [3]. In another study, the amount of MOE was calculated for children 3–6 years old. Its amount was calculated higher than 10,000 and indicated the safety of the bread product for children of this age range [28]. Kamalabadi et al. conducted a study in Iran, calculating the exposure to PAH4 from both industrial and traditional bread (refer to Table 1). For industrial bread, the MOE was deemed acceptable and posed no risk [35]. Furthermore, a study was conducted to assess the risk for B[a]P based on the consumption of 0.3 kg of bread per day and an average weight of 60 kg for people in Nigeria. Daily intake of B[a]P was calculated in the range between 0.01 and 0.19 µg/kg [29]. According to the report of the JECFA Committee (Joint FAO/WHO Expert Committee on Food Additives), the mean daily intake for B[a]P is calculated to be 4 ng/kg bw/day, with a high of 10 ng/kg bw/day (TRS 930-JECFA 64/61). Some manuscripts consider this amount to be 0.5 ng/kg bw/day [3, 55]. However, the authors of this manuscript, based on the Provisional Tolerable Weekly Intake (PTWI) of 7.3 µg/kg for B[a]P, declare the product safe for use [29].

### Analytical method and sample preparation for measuring PAHs

**Fig. 3:**
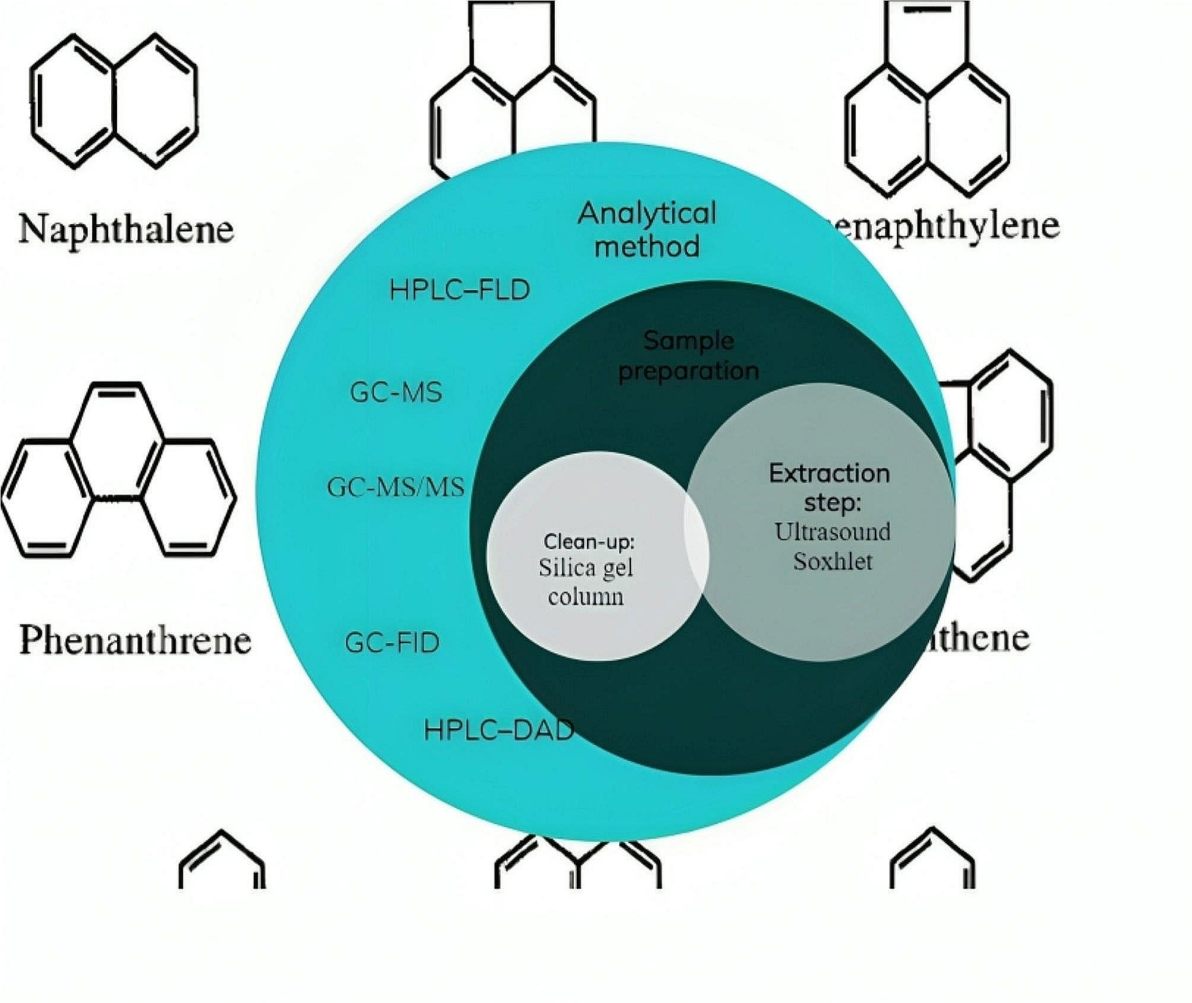
Common methods of determining the amount of PAHs in bread

Sample preparation typically includes extraction and clean-up processes. In order to extract and prepare the samples, a soxhlet was usually used or it was carried out using an ultrasound bath (Fig. 3). In a few studies, both systems were used for extraction [44]. In two studies, QuEChERS(quick easy cheap effective rugged safe) method was used to prepare the samples [27, 36]. It is important to note that one of the reasons for the difference in the amount of PAH4 depends on the method of preparing the samples. For instance, in one study, QuEChERS method was utilized, and in another study, microwave-assisted extraction (MAE) with dispersive liquid–liquid microextraction (DLLME) was used. In the second method, the PAH is concentrated [35]. In a study aimed at optimizing solvents for sample preparation, three solvents—methylene chloride, acetonitrile, and methanol—were used. The formation rate of carbon dioxide bubbles with acetonitrile was lower than the other two types of solvents. The formation of carbon dioxide bubbles can lead to the evaporation of the analyte, subsequently reducing its concentration [39].

The clean-up stage is essential for foods with high fat content [56]. For this purpose, in some studies, a silica gel column has been used [44]. The majority of studies utilized an internal standard. In some studies, certified reference materials have been also used to determine uncertainty.

Based on the extracted data, the majority of analytical measurements were conducted using GC-MS (Table 1). In the case of HPLC measurements, both UV and fluorescence detectors were employed. The mobile phase consisted of a mixture of acetonitrile and water [5]. The gradient method was usually applied for HPLC [3, 5]. In two studies, GC-MS/MS was used for identification and measurement [13, 28]. The advantage of this method is that it requires a very small volume of prepared samples. Furthermore, this analytical method is accurate and has been used in most studies based on extracted data. In a study, GC and HPLC methods were used to determine the amount of PAH in bread. Interestingly, no significant difference was observed between the results obtained from the two methods [3].

One of the most important challenges in determining the amount of PAH in food is the matrix effect. To overcome this challenge, a common approach is to calculate the percent recovery [27, 36]. Typically, samples are spiked, and the recovery percentage is subsequently calculated. It is noteworthy that the recovery percentage was consistently high and deemed acceptable in the majority of studies (Table 1).

## Conclusion

The findings from this systematic review can offer valuable insights into whether humans may be exposed to carcinogenic PAHs through bread consumption. It was observed that, in most cases, there is no significant risk of PAHs contamination for industrial bread. The variation in the PAH4 content among different bread samples is indeed linked to the cooking methods employed. The temperature and duration of cooking directly impact the amount of PAHs present in the bread. Additionally, the type of bread itself contributes to the observed differences in PAH levels. Furthermore, apart from the type of fuel used, the amount of PAHs is influenced by the fat content in the bread. To minimize PAH exposure through bread consumption, it is essential to proactively control and limit these influencing factors. Managing cooking temperatures and durations, selecting specific types of bread, regulating the type of fuel used, and controlling fat content are effective measures in reducing PAH levels in bread. This approach contributes to creating a safer and healthier bread product for consumers. It is worth noting that all the influencing factors on the amount of PAHs in bread have not been investigated. Therefore, other factors that can possibly affect the amount of PAHs, such as the thickness of the bread, are suggested for future research.

### Electronic supplementary material

Below is the link to the electronic supplementary material.

Supplementary Material 1

## Data Availability

The datasets were generated and analyzed during the current study available from the corresponding author on reasonable request.
